# Structural Study of the HD-PTP Bro1 Domain in a Complex with the Core Region of STAM2, a Subunit of ESCRT-0

**DOI:** 10.1371/journal.pone.0149113

**Published:** 2016-02-11

**Authors:** Juhyeon Lee, Kyoung-Jin Oh, Dasom Lee, Bo Yeon Kim, Joon Sig Choi, Bonsu Ku, Seung Jun Kim

**Affiliations:** 1 Disease Target Structure Research Center, Korea Research Institute of Bioscience and Biotechnology, Daejeon, Korea; 2 Department of Biochemistry, Chungnam National University, Daejeon, Korea; 3 Research Center for Metabolic Regulation, Korea Research Institute of Bioscience and Biotechnology, Daejeon, Korea; 4 Incurable Diseases Therapeutics Research Center, World Class Institute, Korea Research Institute of Bioscience and Biotechnology, Ochang, Cheongwon, Korea; Russian Academy of Sciences, Institute for Biological Instrumentation, RUSSIAN FEDERATION

## Abstract

EGFR is a key player in cell proliferation and survival signaling, and its sorting into MVBs for eventual lysosomal degradation is controlled by the coordination of multiple ESCRT complexes on the endosomal membrane. HD-PTP is a cytosolic protein tyrosine phosphatase, and is associated with EGFR trafficking by interacting with the ESCRT-0 protein STAM2 and the ESCRT-III protein CHMP4B via its N-terminal Bro1 domain. Intriguingly, the homologous domain of two other human Bro1 domain-containing proteins, Alix and Brox, binds CHMP4B but not STAM2, despite their high structural similarity. To elucidate this binding specificity, we determined the complex structure of the HD-PTP Bro1 domain bound to the STAM2 core region. STAM2 binds to the hydrophobic concave pocket of the HD-PTP Bro1 domain, as CHMP4B does to the pocket of Alix, Brox, or HD-PTP but in the opposite direction. Critically, Thr145 of HD-PTP, corresponding to Lys151 of Alix and Arg145 of Brox, is revealed to be a determinant residue enabling this protein to bind STAM2, as the Alix- or Brox-mimicking mutations of this residue blocks the intermolecular interaction. This work therefore provides the structural basis for how HD-PTP recognizes the ESCRT-0 component to control EGFR sorting.

## Introduction

Epidermal growth factor receptor (EGFR) is a well-known cell-surface receptor tyrosine kinase, and one of the key regulators of cell survival and growth. Its aberrant expression or uncontrolled activity is directly implicated in a variety of tumors [1]. Binding of ligands, such as epidermal growth factor and transforming growth factor α, to the extracellular domain of EGFR triggers the homodimerization and autophosphorylation of the intracellular domain. A number of downstream signal transduction cascades subsequently initiate, eventually leading to cellular proliferation and differentiation and to the blockade of apoptosis [2,3]. EGFR activity is controlled by clathrin-dependent endocytosis, in which internalized EGFR is recycled back to the plasma membrane or is ubiquitinated and incorporated into intraluminal vesicles (ILVs) in multivesicular bodies (MVBs) for trafficking to lysosomes to be degraded [4,5]. The sorting of ubiquitinated cargo to MVBs is mainly mediated by the endosomal sorting complex required for transport (ESCRT) machinery, which consists of five multiprotein complexes (ESCRT-0, -I, -II, -III and Vps4-Vta1) together with accessory components [6,7]. In addition, a variety of proteins have been reported to interact with the ESCRT proteins to regulate EGFR sorting, such as Alix [8], SARA and RNF11 [9], UBE4E [10], and His domain-containing protein tyrosine phosphatase (HD-PTP; also known as PTPN23) [11].

HD-PTP is a non-receptor type protein tyrosine phosphatase (PTP) that contains five domains (Bro1, V, central proline-rich, PTP and second proline-rich domain). This protein has been reported to negatively regulate endothelial cell motility [12,13] and to function as a putative tumor suppressor [14,15]. Considerable evidence has also indicated that HD-PTP and Myopic, the homologue of HD-PTP in *Drosophila melanogaster*, are involved in the morphogenesis of MVBs and in the endosomal sorting of cargo proteins, including integrin [16], Wntless and Wingless [17], and EGFR [11,18], and that the Bro1 domain is necessary for these processes. However, the precise relationship between PTP enzymatic activity, tumor-suppressive capacity, and trafficking-regulating effect of HD-PTP remains to be elucidated.

Thus far, two ESCRT components have been found to interact with the Bro1 domain of HD-PTP: the ESCRT-0 protein signal-transducing adaptor molecule 2 (STAM2) [11] and the ESCRT-III protein charged multivesicular body protein 4B (CHMP4B) [19,20]. STAM2 binds hepatocyte growth factor-regulated tyrosine kinase substrate (Hrs) to form the ESCRT-0 heterodimer, which initiates the MVB pathway with its multiple ubiquitin-binding domains clustering ubiquitinated EGFR at the endosomal membrane [7,21]. CHMP4B is one of the core subunits of the ring-shaped ESCRT-III filament that carries out membrane remodeling through budding and scission, which is critical for the formation of mature ILVs [21,22]. A recent report indicated that HD-PTP is the central regulator of EGFR sorting that functions to coordinate the transfer of EGFR from STAM2-containing ESCRT-0 to CHMP4B-containing ESCRT-III [11]. Herein, we identified that the residues 350–370 of STAM2 are necessary and sufficient for directly binding the Bro1 domain of HD-PTP, and determined the structure of the complex to a resolution of 2.0 Å. Structural alignments and structure-based mutant studies together led to the discovery of a key determinant residue, Thr145 in HD-PTP corresponding to Lys151 in Alix and Arg145 in Brox, that defines the STAM2-binding selectivity favoring the Bro1 domain of HD-PTP over those of Alix and Brox.

## Results

### Elucidation of the HD-PTP Bro1 Domain-Binding Region in STAM2

**Fig 1:**
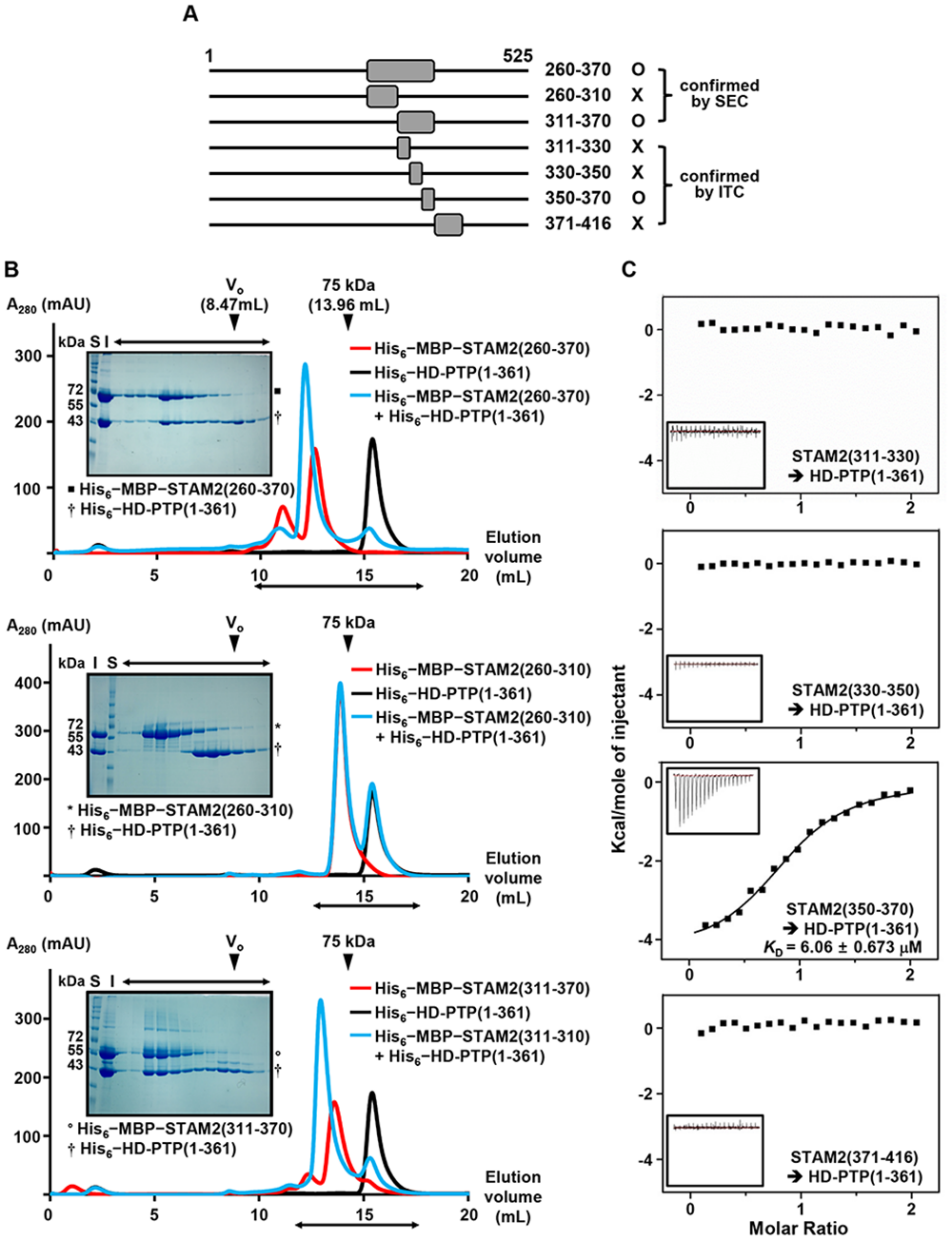
Interaction of HD-PTP(1–361) with the STAM2 core region. (A) STAM2 constructs tested for binding to HD-PTP(1–361). Denoted beside the residue numbers is whether each construct interacted with HD-PTP(1–361) (B) SEC analysis results using a Superose 6 10/300 GL gel filtration column. The elution positions of standard protein size markers Blue dextran (void volume, V_0_) and Conalbumin (75 kDa) are indicated by arrowheads. The proteins tested in each analysis are denoted (*Right*). The peak fractions from the HD-PTP and STAM2 mixture elution were analyzed and visualized by SDS-PAGE and Coomassie staining (*Left*). S, size marker; I, input. (C) ITC analysis. Each 0.5 mM STAM2 peptide was titrated into 50 μM HD-PTP(1–361). The *K*_D_ value was deduced from curve fittings of the integrated heat per mole of added ligand.

Previously, the Bro1 domain of HD-PTP was reported to interact with the core region of STAM2 compromising residues 260–416, as demonstrated by yeast two-hybrid and coimmunoprecipitation assays [11]. To verify the direct binding between the two proteins, we prepared the recombinant HD-PTP Bro1 domain containing residues 1–361 (referred to as HD-PTP(1–361)) and the STAM2 core region containing residues 260–370 (referred to as STAM2(260–370)) (Fig 1A). We excluded residues 371–416 of STAM2 because these residues were predicated not to form a secondary structure (S1 Fig) and thus were speculated not to be involved in the binding interaction with HD-PTP(1–361). Size-exclusion chromatography (SEC) analysis revealed that these two proteins indeed directly interact with each other (Fig 1B, top panel). To elucidate the precise binding region, we divided STAM2(260–370) into two fragments, STAM2(260–310) and STAM2(311–370) (Fig 1A). Ensuing chromatography analysis determined that HD-PTP binds to STAM2(311–370) but not STAM2(260–310) (Fig 1B, middle and bottom panels). Using isothermal titration calorimetry (ITC), we next examined and quantified the interaction of HD-PTP(1–361) with three STAM2 peptides containing the residues 310–330, 330–350 and 350–370 of STAM2, respectively (Fig 1C). The residues 350–370 of STAM2, referred to as STAM2(350–370), were identified to be necessary and sufficient for binding HD-PTP(1–361), with a resulting *K*_D_ value of 6.06 μM (Fig 1C, third panel). We also confirmed using ITC that the STAM2 peptide compromising residues 371–416 is unable to bind HD-PTP(1–361) (Fig 1C, fourth panel).

### Structure Determination of the HD-PTP Bro1 Domain Bound to the STAM2 Fragment

**Fig 2:**
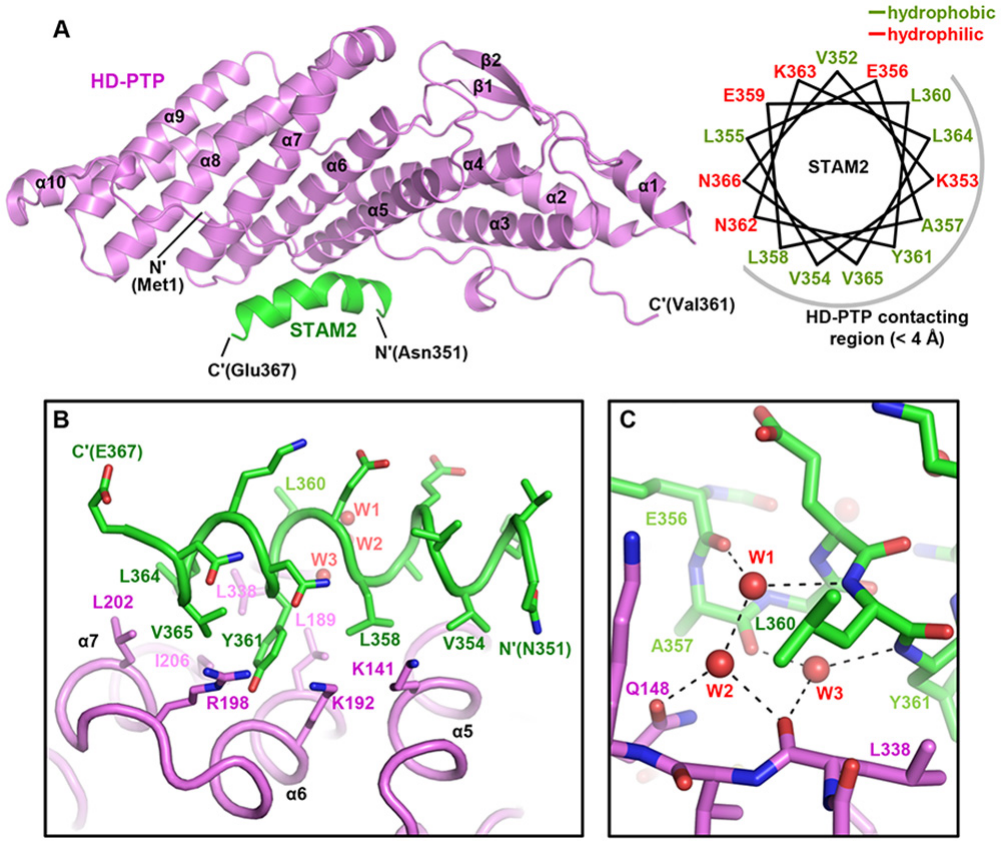
Structural analysis of the interaction between HD-PTP and STAM2. (A) Crystal structure of the HD-PTP(1–361;NAYA)−STAM2(350–370) complex. (*Left*) The two proteins are presented as ribbon drawings with the labels of secondary structures according to the order of their appearance in the primary sequence. (*Right*) α-helical wheel representation of the STAM2 fragment shown in the complex structure. STAM2 residues in contact with those of HD-PTP within 4 Å are covered by a gray semicircle. (B-C) Intermolecular hydrophobic interaction (*B*) and water-mediated hydrogen bonds (*C*). Shown in sticks are all STAM2 residues together with the HD-PTP residues involved in the complex formation step. Hydrogen bonds mediated by three water molecules (shown in red sphere) are represented as dotted lines. Labeled are the two protein residues participating in the intermolecular interaction.

Based on this result, we subsequently attempted to determine the complex structure of HD-PTP(1–361) bound to the STAM2(350–370) peptide. Our initial trial was unsuccessful, because even though the protein and the peptide were mixed and incubated at a 1:5 molar ratio before crystallization, the resulting crystals (space group *P*1) contained four HD-PTP(1–361) molecules in the asymmetric unit but did not contain STAM2(350–370), which was revealed after the structure determination (S2A and S2B Fig; PDB code 5CRU). All four HD-PTP(1–361) monomers are very well matched with the previously determined crystal structure of the HD-PTP Bro1 domain (PDB code 3RAU) [20] with a root-mean-square deviation (RMSD) value in the range of 0.48–0.77 Å. It was previously indicated that the binding of the HD-PTP Bro1 domain to STAM2 can be compromised via an aspartate substitution of Leu202 and Ile206, implying that these residues play a role in the binding interaction [11]. We noticed that in our HD-PTP(1–361) structure, these residues (together with neighboring residues Arg205, Ala336, and Leu338) form hydrophilic and hydrophobic contacts with Asn33 and Tyr34 from an adjacent HD-PTP monomer (S2C Fig), suggesting that the crystal packing interaction between HD-PTP molecules will prevent STAM2(350–370) from being accommodated in HD-PTP(1–361). Therefore, with the expectation of altering crystal packing and promoting crystallization in a STAM2-bound form, we prepared a mutant HD-PTP(1–361) protein in which Asn33 and Tyr34 are substituted for alanine (referred to as HD-PTP(1–361;NAYA)). This mutant protein binds STAM2(350–370) as potently as the wild-type protein; this was confirmed using ITC (S3A Fig). After incubation at a molar ratio of 1:5 between HD-PTP(1–361;NAYA) and the STAM2(350–370) peptide overnight, we crystallized the protein sample and obtained novel crystals with the space group *P*2_1_ containing two HD-PTP molecules in the asymmetric unit. Using these crystals, we finally determined the crystal structure of HD-PTP(1–361;NAYA) in a complex with STAM2(350–370) to 2.0 Å (Fig 2 and S4 Fig; PDB code 5CRV). We note that both the interaction of the STAM2 peptide and the insertion of the N33A and Y34A mutations do not induce a dramatic conformational change of the HD-PTP Bro1 domain, as our HD-PTP(1–361) and HD-PTP(1–361;NAYA) structures overlap each other greatly when superposed with a RMSD value of 0.61 Å over 355 aligned residues. Nevertheless, together with a slight conformational change of α1-α2 loop (S5A Fig), a remarkable difference in the arrangement of the HD-PTP molecules was shown in the two crystal forms (S5B Fig), which allows STAM2(350–370) to be accommodated in HD-PTP(1–361;NAYA) and the complex structure to be determined. Crystallographic data statistics are summarized in Table 1.

**Table 1:** Data collection and structure refinement statistics.

Data Collection	HD-PTP(1–361)	HD-PTP(1–361;NAYA)–STAM2(350–370)
Space group	*P*1	*P*2_1_
Unit cell dimensions		
a, b, c (Å)	64.99, 66.87, 82.42	81.65, 37.22, 140.02
α, β, γ (°)	89.9, 89.9, 89.8	90, 103.4, 90
Wavelength (Å)	0.9795	0.9795
Resolution (Å)	50.0–2.4 (2.44–2.40)	50.0–2.0 (2.03–2.00)
*R*_sym_ (%)	9.4 (60.2)	6.4 (26.3)
*I*/σ(*I*)	24.6 (3.3)	30.4 (4.6)
Completeness (%)	98.3 (97.9)	97.3 (95.5)
Redundancy	3.8	4.8
**Refinement**		
Resolution (Å)	50.0–2.4	50.0–2.0
No. of reflections	50948	54756
*R*_work_ / *R*_free_ (%)	22.0 / 25.9	21.3 / 24.1
RMSD		
Bond lengths (Å)	0.005	0.003
Bond angles (°)	1.012	0.701
Average B-values (Å^2^)	46.5	34.3
Ramachandran plot (%)		
Most favored	98.4	96.9
Additionally allowed	1.6	3.1

The numbers in parentheses are statistics from the shell with the highest resolution.

### Analysis of the Interaction between HD-PTP and STAM2

Alix, Brox and HD-PTP are three human proteins containing a Bro1 domain that binds the ESCRT-III component CHMP4B, as is well established by means of structural determination and complex modeling studies [20,23,24]. Similar to that of Alix or Brox, the Bro1 domain of HD-PTP adopts a boomerang-like fold containing a concave binding pocket, where the C-terminal tail of CHMP4 may be accommodated [20]. In the HD-PTP(1–361;NAYA)−STAM2(350–370) complex structure, the STAM2 peptide forms an amphipathic α-helix that binds to HD-PTP, mainly through hydrophobic interaction (Fig 2A and 2B and S6 Fig). In detail, the intermolecular hydrophobic interactions involve Val354, Leu358, Tyr361, Leu364 and Val365 of STAM2 and Leu189, Leu202, Ile206, Ala336, Leu338 and the hydrocarbon portions of Lys141, Lys192, and Arg198 of HD-PTP. Specifically, Tyr361 of STAM2 is located at the center of the hydrophobic cluster (Fig 2B and S6 Fig), suggesting that this residue plays a key role in the complex formation. Alanine substitution of this residue completely abrogated the binding interaction between the two proteins, as confirmed by ITC measurements (S3B Fig). The involvement of Leu202 and Ile206 of HD-PTP in the hydrophobic interaction is in good agreement with the previous finding from a mutational study which showed that aspartate substitutions of these residues (the L/I-D/D mutation) impaired the complex formation [11]. Along with the hydrophobic interactions, we note that hydrogen bonds mediated by three water molecules also reinforce the intermolecular binding between HD-PTP and STAM2 (Fig 2C).

### Thr145 Is a Key Determinant of HD-PTP in Binding STAM2

**Fig 3:**
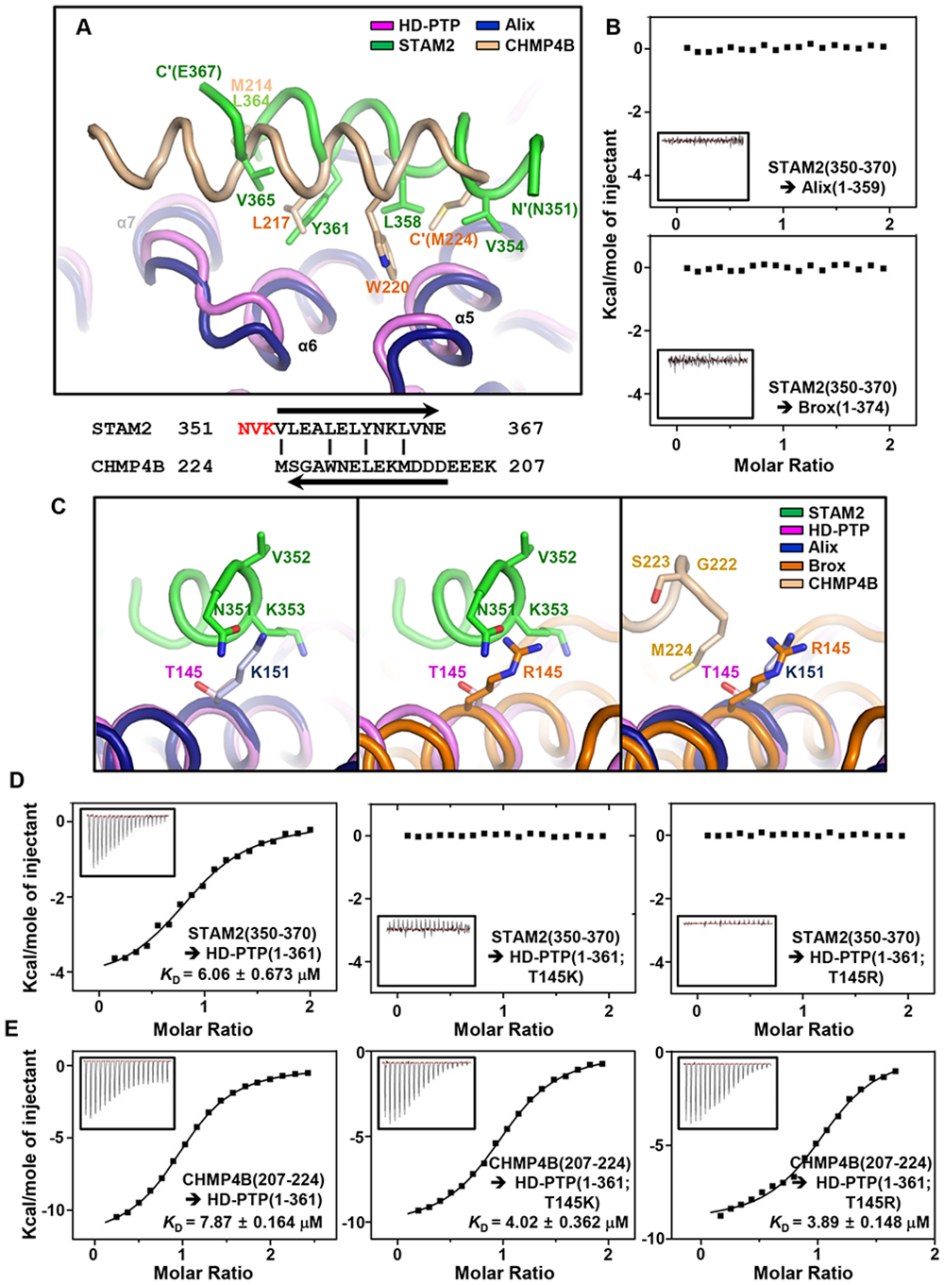
Structural analysis of the binding selectivity of three Bro1 domains. (A) Structural comparison between HD-PTP(1–361;NAYA)−STAM2(350–370) and Alix(1–359)−CHMP4B(207–224) (PDB code 3C3Q) complexes. The key residues in the intermolecular hydrophobic interactions are shown in sticks and are labeled. The STAM2 and CHMP4B residues are reverse-aligned below, with the vertical lines matching the labeled residues. (B) Neither the Bro1 domain of Alix nor Brox binds STAM2(350–370). ITC measurements were carried out by titrating the 0.5 mM STAM2(350–370) peptide into the 50 μM Alix(1–359) and Brox(1–374) proteins. (C) Thr145 is the key residue in HD-PTP binding to STAM2. The HD-PTP(1–361;NAYA)−STAM2(350–370) structure is superposed on the Alix(1–359)−CHMP4B(207–224) (*Left*) and the Brox(1–377)−CHMP4B(207–224) (PDB code 3UM3) (*Middle*) complexes. Lys151 of Alix and Arg145 of Brox cause steric hindrance with STAM2, but Thr145 of HD-PTP does not. (*Right*) None of the three Bro1 domain residues brings about steric hindrance with CHMP4B. (D) Mutation of Thr145 prevents HD-PTP(1–361) from binding STAM2(350–370). ITC measurements were performed by titrating the 0.5 mM STAM2(350–370) peptide into 50 μM HD-PTP proteins. The left graph showing the interaction between STAM2(350–370) and HD-PTP(1–361) is identical to that in Fig 1C, which is included in this figure for comparison. (E) CHMP4B(207–224) interacts with HD-PTP(1–361) regardless of the Thr145 residue mutations. The 0.5 mM CHMP4B(207–224) peptide was titrated into 50 μM HD-PTP proteins, and the *K*_D_ values were deduced.

At a glance, CHMP4B(207–224) and STAM2(350–370) bind to the concave pocket of the Bro1 domain in a similar manner, mainly through their hydrophobic residues. We therefore superposed our HD-PTP(1–361;NAYA)−STAM2(350–370) complex structure onto the structure of the Bro1 domain of Alix bound to the CHMP4B peptide (residues 207–224; referred to as CHMP4B(207–224)). Indeed, STAM2(350–370) and CHMP4B(207–224) overlap when bound to the Bro1 domains, and the hydrophobic residues of the two proteins involved in the intermolecular interaction can be matched one by one (Fig 3A). Thus, the ESCRT-III component CHMP4B should compete with and displace the ESCRT-0 component STAM2 from the Bro1 domain of HD-PTP, as previously suggested [11]. One notable difference between STAM2(350–370) and CHMP4B(207–224) is that they associate with the Bro1 domains in opposite orientations (Fig 3A, bottom). Moreover, while the residues 354–367 of STAM2 are able to correspond to the residues 211–224 of CHMP4B, the preceding residues 351–353 of STAM2 do not, as the CHMP4B polypeptide terminates with Met224 matched to Val354 of STAM2 (Fig 3A).

Despite the overall structural similarity among the Bro1 domains, a recent report by Ali *et al*. indicated that the Bro1 domain of Alix was not coprecipitated with STAM2 unlike that of HD-PTP [11]. We thus verified the interaction between the Bro1 domains and the core region of STAM2 using ITC. Indeed, unlike HD-PTP(1–361), neither the Bro1 domains of Alix (residues 1–359) nor Brox (residues 1–374) interact with STAM2(350–370) (Fig 3B), despite the fact that the key residues of HD-PTP in the binding to STAM2 are mostly conserved in Alix and Brox (S7 Fig). We therefore looked into the superposed complex structures. Intriguingly, we found that the STAM2 residues 351–353 play a key role in determining the binding partner of the protein; in the superposed models, this region brings about steric hindrance with the side chains of Lys151 of Alix and Arg145 of Brox, but not with that of the corresponding residue Thr145 of HD-PTP (Fig 3C; left and middle panels). This threonine residue at first did not appear as a key residue in the intermolecular interaction of HD-PTP with STAM2 in the complex formation. Indeed, the alanine substitution of Thr145 of HD-PTP(1–361) did not abolish its binding to STAM2(350–370) (S3C Fig). Nevertheless, structural superposition analysis revealed a possibility that the presence of threonine at that position in HD-PTP, instead of lysine as in Alix and arginine as in Brox, would facilitate the complex formation by “avoiding” or “not making” steric hindrance with the STAM2 helix. To corroborate this hypothesis, we prepared two mutant HD-PTP(1–361) proteins: Alix-mimicking HD-PTP(1–361;T145K) and Brox-mimicking HD-PTP(1–361;T145R). In the ITC experiments, neither HD-PTP(1–361;T145K) nor HD-PTP(1–361;T145R) interacted with the STAM2(350–370) peptide (Fig 3D), demonstrating the significance of the steric clash we found in the interaction between STAM2 and the Bro1 domains. Otherwise, CHMP4B does not contain residues corresponding to the STAM2 residues 351–353 causing steric hindrance (Fig 3C; right panel). We thus predicted that the mutation of Thr145 would not affect the interaction between HD-PTP and CHMP4B. This was also confirmed using ITC; CHMP4B(207–224) bound well to wild type and the HD-PTP mutant proteins (*K*_D_ values of less than 8 μM; Fig 3E and S3D Fig) as anticipated. Collectively, these results demonstrate that Thr145 is a unique functional determinant residue of HD-PTP, which enables the protein to bind to the core region of STAM2 without steric hindrance.

**Fig 4:**
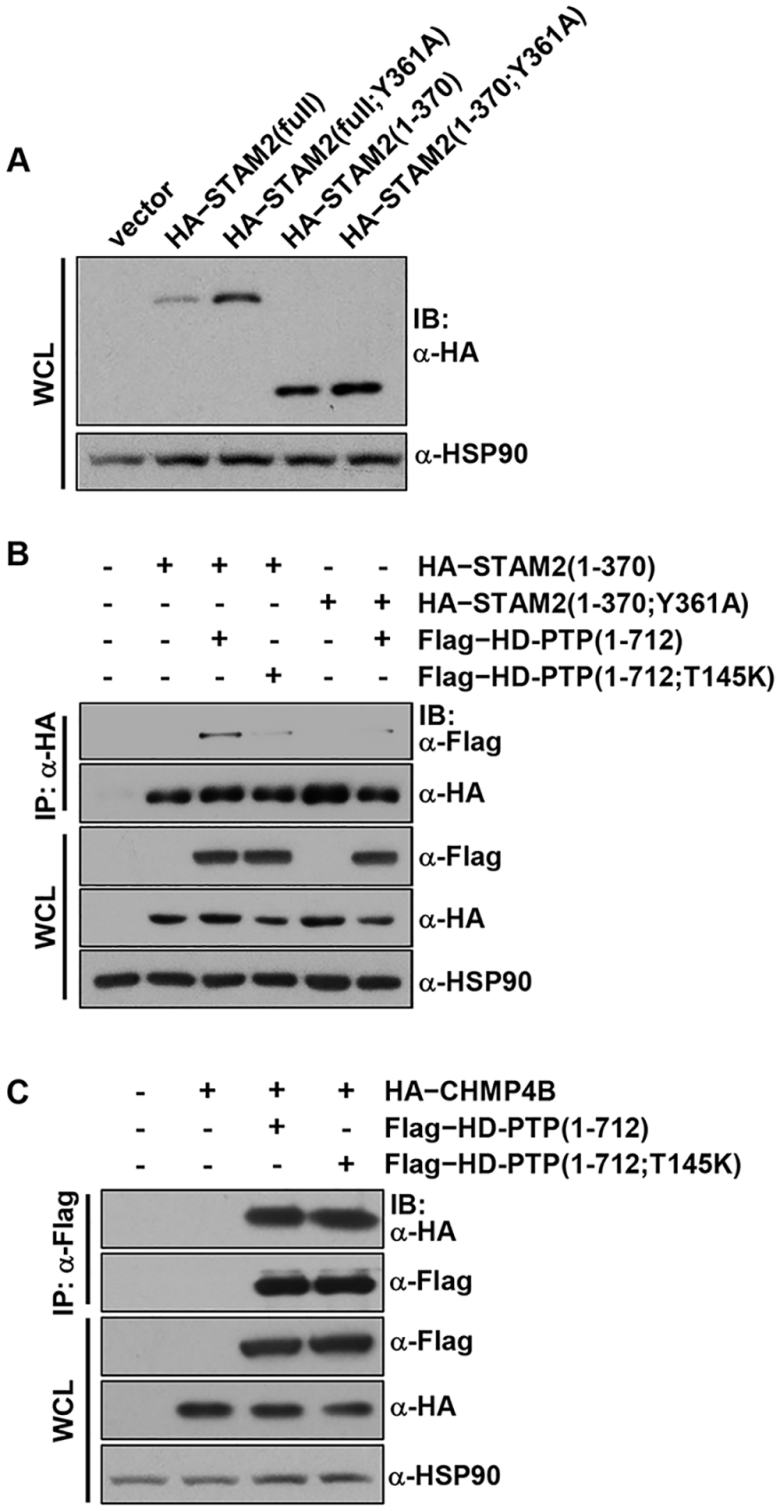
Coimmunoprecipitation assay between HD-PTP and STAM2 or CHMP4B. HEK293 cells were transiently transfected with the indicated constructs, and the expression level of STAM2 proteins (*A*), or intermolecular interaction of HD-PTP with STAM2 (*B*) or with CHMP4B (*C*) was assessed by immunoprecipitation and immunoblotting.

Next, the interactions in human cells between STAM2, CHMP4B, and the Bro1 domain of HD-PTP were confirmed by pull-down assays. In order to concentrate on the Bro1 domain-mediated intermolecular association and to exclude the effect of the additional binding between the SH3 domain of STAM2 and the central proline-rich domain of HD-PTP [11], Flag-tagged HD-PTP(1–712) variants together with HA-tagged CHMP4B and STAM2 proteins were transiently expressed in human embryonic kidney 293 (HEK293) cells, and immunoprecipitation assays were performed. Due to low expression level of full-length STAM2 (Fig 4A), STAM2(1–370) and STAM2(1–370;Y361A) were subjected to immunoprecipitation. The results indicated that STAM2 indeed interacted with HD-PTP(1–712) but only very weakly reacts with HD-PTP(1–712;T145K) (Fig 4B; third and fourth columns), demonstrating the significance of the residue Thr145 of HD-PTP in binding to STAM2. In contrast, CHMP4B physically interacted with HD-PTP(1–712;T145K) as well as HD-PTP(1–712) (Fig 4C), which is consistent with our structural analysis and binding measurements. We also confirmed that the STAM2 binding to HD-PTP is abrogated by the alanine substitution of Tyr361 of STAM2 (Fig 4B; third and sixth columns), the key residue of the intermolecular hydrophobic interaction between the two proteins (see Fig 2B and S6 Fig).

## Discussion

As previous studies have indicated, the boomerang-shaped Bro1 domain of HD-PTP shares considerable sequence and structural similarity with that of Alix or Brox (S7 Fig, bottom table). Together with their structural similarity, all the three Bro1 domain-containing proteins are known to interact with the ESCRT-III component CHMP4B through their concave pocket. Although the role of Brox in protein sorting is not yet well defined, both HD-PTP and Alix were determined to be involved in the EGFR sorting to the MVB [8,11]. Nevertheless, despite such similarities, Alix, Brox and HD-PTP also have their own unique structural features that enable the three proteins to function differently and specifically. For instance, only the Bro1 domain of Alix functions during the release of human immunodeficiency virus-1 (HIV-1), despite the fact that all three are able to interact with the nucleocapsid domain of the Gag protein of HIV-1 [25,26]. Structural studies revealed that this is due to a distinguishing Phe105 loop only present in the Bro1 domain of Alix, which is essential for the HIV-1 release [20,27]. On the other hand, the C-terminal tail of CHMP5 binds to the Bro1 domain of Brox but not to that of Alix or HD-PTP, in which nonconserved Tyr348 of Brox plays a critical structural role in constituting a unique binding pocket for the β-hairpin structure of CHMP5 [24]. Likewise, our structural and biochemical studies provide a rational explanation of the binding selectivity of STAM2 to the Bro1 domain of HD-PTP over that of Alix or Brox; the avoidance of steric hindrance due to the presence of a threonine residue instead of lysine or arginine is the key feature of the HD-PTP Bro1 domain, enabling this protein to accommodate STAM2 in its binding pocket.

In HD-PTP and Alix, but not in Brox, the Bro1 domain is followed by a V domain (residues 362–699 of HD-PTP). The two V domains share 17% sequence identity and 45% similarity with each other, and both were reported to interact with the Lys63-linked polyubiquitin chain [28–30]. The V domain of Alix is also known to provide a binding module for the YPX_3_L motif of protease-activated receptor 1 [31], the YPX_n_L late-domain motif of the p6 domain of the Gag protein of HIV and other viruses that require it for viral budding [32,33]. The residues in the V domain of Alix that associate with those motifs are mostly conserved in that of HD-PTP as well, including the key phenylalanine residue (Phe676 of Alix; Phe678 of HD-PTP) [34]. Interestingly, a study by Stefani *et al*. addressed that ubiquitin-associated protein 1 (UBAP1), an ESCRT-I component involved in EGFR sorting to the MVB, binds the V domain of HD-PTP but not the corresponding domain of Alix [35], providing another case of an ESCRT protein that selectively binds HD-PTP over Alix. Aspartate substitution of Phe678 of HD-PTP abolished its interaction with UBAP1, suggesting that the UBAP1-binding region might overlap with the presumed YPX_*n*_L motif-binding region in HD-PTP. We thus consider that, as in case of the HD-PTP Bro1 domain and the ESCRT-0 component STAM2, structural study of the V domain of HD-PTP would be necessary to elucidate the basis of its selective binding to UBAP1, which might also contribute to the understanding of the precise role and function of HD-PTP in EGFR sorting

In this work, we discovered that the residues 350–370 of STAM2 constitute the HD-PTP-binding region, and presented the crystal structure of the Bro1 domain of HD-PTP in a complex with the core fragment of STAM2. We further delineated the structural feature of the Bro1 domain of HD-PTP, discriminating it from that of Alix or Brox, as certified by structural analyses, ITC binding measurements, and coimmunoprecipitation assays. We believe that this structural information will be a rational basis for future investigations to unravel the overall working mechanism of EGFR trafficking, which has received great interest due to its direct association with cell survival and cancer signaling.

## Materials and Methods

### Preparation, Crystallization, and Structure Determination of HD-PTP(1–361) and HD-PTP(1–361;NAYA) Bound to STAM2(350–370)

The DNA fragment coding for the Bro1 domain of human HD-PTP (residues 1–361) was amplified by a polymerase chain reaction and cloned into the pET28a plasmid (Novagen), which was used as the template for the preparation of the mutant form of HD-PTP containing N33A and Y34A substitutions. Wild type or mutant HD-PTP protein was produced in the *E*. *coli* BL21(DE3) RIL strain (Novagen) at 18°C and initially purified using a Ni-NTA column (QIAGEN). After the removal of the N-terminal (His)_6_-tag through a TEV protease treatment, the protein was further purified using a HiPrep 26/60 Sephacryl S-100 HR gel filtration column (GE Healthcare) equilibrated with a buffer solution containing 20 mM Tris-HCl (pH 7.5), 200 mM NaCl, 10% (v/v) glycerol and 10 mM dithiothreitol. The final HD-PTP protein samples were mixed at a 1:5 molar ratio with the 20-mer STAM2 peptide (residues 350–370) purchased from Peptron (Korea). HD-PTP(1–361) crystals were obtained by the sitting-drop vapor diffusion method at 18°C by mixing and equilibrating a 0.2 μL protein solution (33 mg/mL) and a 0.3 μL precipitant solution containing 0.03 M HEPES sodium (pH 7.3), 16% (w/v) polyethylene glycol 3350, 0.67% (w/v) tryptone and 0.03 M urea. Before the data collection process, HD-PTP(1–361) crystals were immersed briefly in a cryoprotectant solution, which was identical to the reservoir solution but with 42% (w/v) polyethylene glycol 3350. HD-PTP(1–361;NAYA)−STAM2(350–370) crystals were obtained by the sitting-drop vapor diffusion method at 18°C by mixing and equilibrating a 0.2 μL protein solution (24 mg/mL) and a 0.8 μL precipitant solution containing 0.1 M Bis-Tris (pH 7.0), 30% (w/v) polyethylene glycol 3350 and 4% (v/v) acetonitrile. Before data collection, the complex crystals were immersed briefly in a cryoprotectant solution, which was the reservoir solution plus 5% glycerol. Diffraction data were collected on the beamline 5C at the Pohang Accelerator Laboratory, Korea, and processed using the program *HKL* 2000 [36]. The structures were determined by the molecular replacement method with the program Phaser [37] using the structure of the Bro1 domain of HD-PTP [20] as a search model. The programs Coot [38] and PHENIX [39] were used for model building and refinement, respectively. Crystallographic data statistics are summarized in Table 1.

### Preparation of Recombinant Proteins and Peptides

Each of the DNA fragments coding for the core region of human STAM2 (residues 260-370/260-310/311-370) were cloned into the pET28a plasmid, designed to produce a protein N-terminally fused to maltose-binding protein together with a (His)_6_-tag. Each of the DNA fragments coding for the Bro1 domain of HD-PTP containing the T145K, T145R or T145A substitutions, Alix (residues 1–359), and Brox (residues 1–374) were cloned into the pET28a plasmid. The proteins were produced in the *E*. *coli* BL21(DE3) RIL strain at 18°C and purified using a Ni-NTA column and a HiPrep 26/60 Sephacryl S-100 HR gel filtration column. Synthetic STAM2 peptides of 21-mer (residues 330-350/350-370/350-370 with Y391A substitution), 20-mer (residues 311–330), and 46-mer (residues 371–416) and a CHMP4B peptide of 18-mer (residues 207–224) were purchased from Peptron (Korea).

### Isothermal Titration Calorimetry

All measurements were carried out at 2°C on an iTC200 microcalorimetry system (GE Healthcare). Protein samples were dialyzed against the solution containing 20 mM Tris-HCl (pH 7.5) and 100 mM NaCl. The samples were centrifuged to remove any residuals prior to the measurements. Dilution enthalpies were measured in separate experiments (titrant into buffer) and subtracted from the enthalpies of the binding between the protein and the titrant. Data were analyzed using the Origin software (OriginLab Corp.).

### Immunoblotting and Immunoprecipitation

Each of case of the DNA fragment coding for HA–STAM2(full), HA–STAM2(1–370), HA–CHMP4B and Flag–HD-PTP(1–712) were amplified by a polymerase chain reaction with cloning into pcDNA3.1/FRT/TO (Invitrogen). HA–STAM2(full;Y361A), HA–STAM2(1–370;Y361A) and Flag–HD-PTP(1–361;T145K) were generated by site-directed mutagenesis. HEK293 cells (ATCC) transfected with these constructs were harvested after 48 hours and lysed in a buffer containing 50 mM Tris-HCl (pH 7.5), 150 mM NaCl, 1% Nonidet P40, 0.5% sodium deoxycholate and 1mM of EDTA supplemented with a complete proteinase inhibitor cocktail (Roche). Proteins from the cell extracts were immunoprecipitated with anti-HA agarose (Sigma Aldrich) or anti-Flag M2 agarose (Sigma Aldrich). Immunoprecipitated proteins were analyzed by western blot assay using anti-HA (1:5000) (Santa Cruz) and anti-Flag M2 (1:5000) (Sigma Aldrich). An antibody against HSP90 (1:5000) (Santa Cruz) was used to ensure equal loading. The specific protein bands were visualized by enhanced chemiluminescence detection (Amersham).

### Accession Numbers

The coordinates of HD-PTP(1–361) and HD-PTP(1–361;NAYA) in a complex with STAM2(350–370) together with the structure factors have been deposited in the Protein Data Bank with the accession codes of 5CRU and 5CRV, respectively.

## Supporting Information

S1 Fig Secondary structure prediction of the core region of STAM2. This prediction was obtained from PSI-PRED server (http://bioinf.cs.ucl.ac.uk/psipred/). STAM2 residues Leu260, Val370 and Gln416 are indicated by red triangles.
(TIF)

S2 Fig Crystal structure of HD-PTP(1–361). (A) The structure of HD-PTP(1–361) presented as a ribbon drawing. Labels of secondary structures are represented according to the order of their appearance in the primary sequence. (B) C_α_ traces of four molecules of HD-PTP(1–361) in the asymmetric unit of crystals with the space group *P*1. (C) Crystal packing interactions. Asn33 and Tyr34 from one molecule (shown in orange) are in contact with Arg205 and four hydrophobic residues (Leu202, Ile206, Ala336, and Leu338) from an adjacent molecule (shown in light blue). Dashed circle highlights intermolecular hydrophobic interactions.
(TIF)

S3 Fig ITC measurements. ITC measurements were carried out by titrating the 0.5 mM indicated peptide into the 50 μM wild type (*B*) or mutant (*A*, *C*-*D*) HD-PTP proteins.
(TIF)

S4 Fig Stereo 2Fo-Fc omit map of STAM2(350–370). The STAM2(350–370) fragment in the Fig 2B is presented in sticks together with the 2Fo-Fc electron density omit map (grey mesh; contoured at 1.0 σ).
(TIF)

S5 Fig Comparison of HD-PTP(1–361) and HD-PTP(1–361;NAYA) molecules. (A) Conformational change of HD-PTP induced by the introduction of two mutations. The two residues targeted for mutation are shown in sticks and are labeled. The dashed line indicates the movements of the C_α_ atoms of Tyr34 substituted to alanine. (B) Packing of HD-PTP molecules in crystals. HD-PTP(1–361) and HD-PTP(1–361;NAYA) are shown in C_α_ traces. For clarity, STAM2(350–370) is omitted. Mol A and B of HD-PTP(1–361) are in the same asymmetric unit; Mol A, A' and A'' of HD-PTP(1–361;NAYA) are not, but are symmetrically related.
(TIF)

S6 Fig Intermolecular hydrophobic interaction. HD-PTP(1–361;NAYA) is represented in the surface model bound to the STAM2 fragment shown in green. Residues engaged in the intermolecular hydrophobic contacts (five from STAM2 and seven from HD-PTP; see Fig 2B) are labeled. Shown in stick representation are the labeled STAM2 residues; colored in yellow are the hydrocarbon portions of the side chain of the labeled HD-PTP residues.
(TIF)

S7 Fig Structure-based sequence alignment. The sequences of the Bro1 domains of HD-PTP, Alix, and Brox are aligned based on a structural comparison. The secondary structures of HD-PTP are shown together. Conserved residues are shaded in cyan. The key residues adjusting STAM2 binding are shown in red and are highlighted by a black rectangle. Asterisks denote the HD-PTP residues involved in STAM2 binding. The sequence and structural alignment statistics are listed below.
(TIF)
