# Supplementary figures and images for: Structural Study of the HD-PTP Bro1 Domain in a Complex with the Core Region of STAM2, a Subunit of ESCRT-0
Source: PLoS One. 2016 Feb 11;11(2):e0149113. doi: 10.1371/journal.pone.0149113 (PMC4751086; doi:10.1371/journal.pone.0149113)

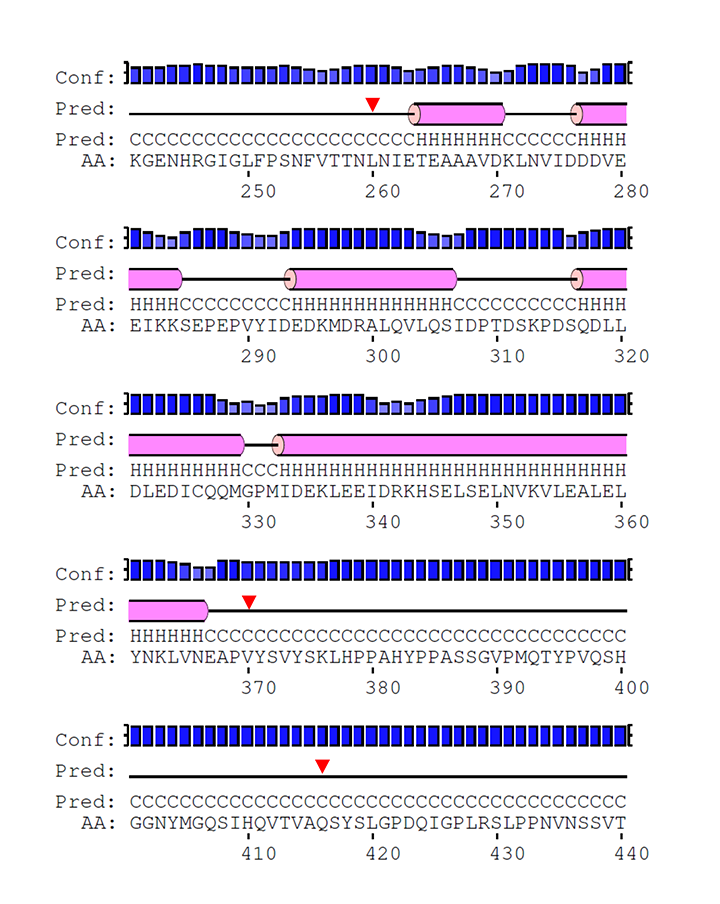

Supplement: S1 Fig — This prediction was obtained from PSI-PRED server (http://bioinf.cs.ucl.ac.uk/psipred/). STAM2 residues Leu260, Val370 and Gln416 are indicated by red triangles. (TIF) [file pone.0149113.s001.tif]

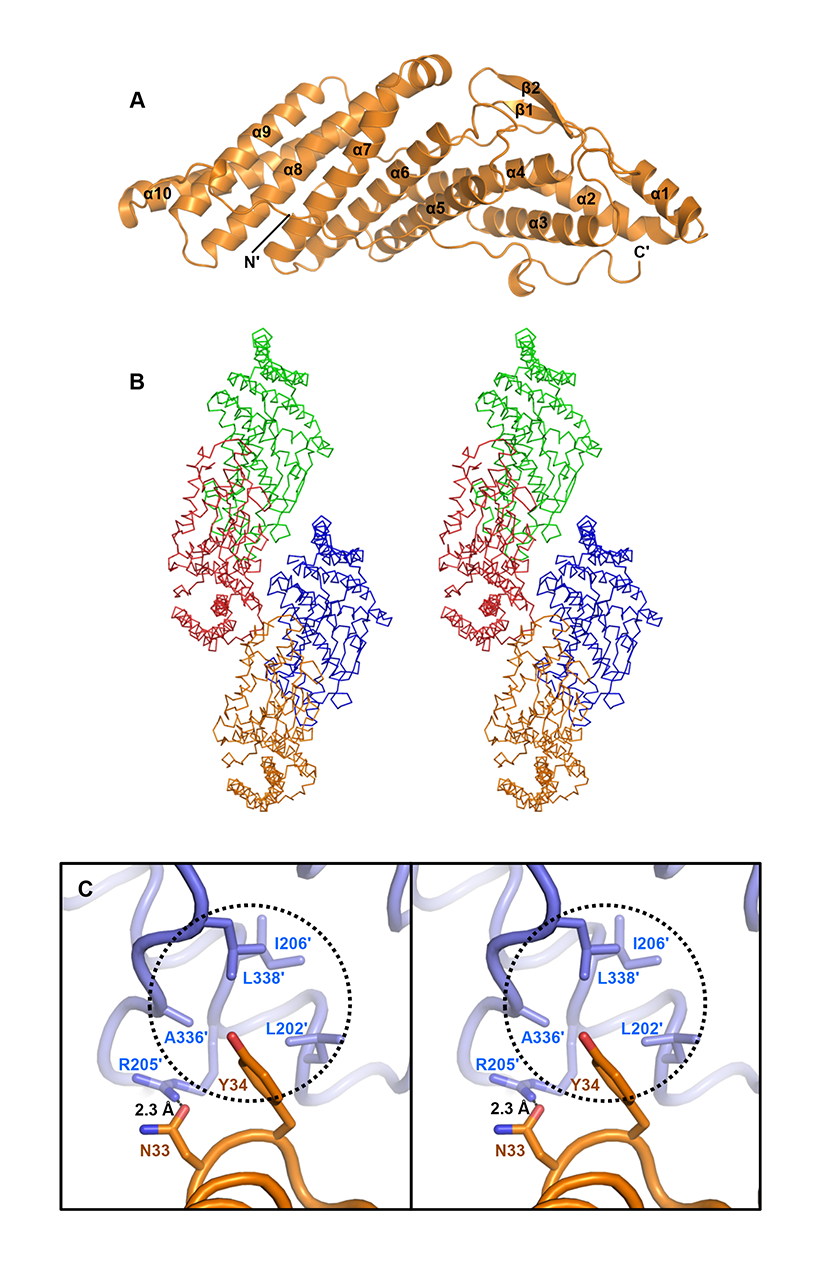

Supplement: S2 Fig — (A) The structure of HD-PTP(1–361) presented as a ribbon drawing. Labels of secondary structures are represented according to the order of their appearance in the primary sequence. (B) Cα traces of four molecules of HD-PTP(1–361) in the asymmetric unit of crystals with the space group P1. (C) Crystal packing interactions. Asn33 and Tyr34 from one molecule (shown in orange) are in contact with Arg205 and four hydrophobic residues (Leu202, Ile206, Ala336, and Leu338) from an adjacent molecule (shown in light blue). Dashed circle highlights intermolecular hydrophobic interactions. (TIF) [file pone.0149113.s002.tif]

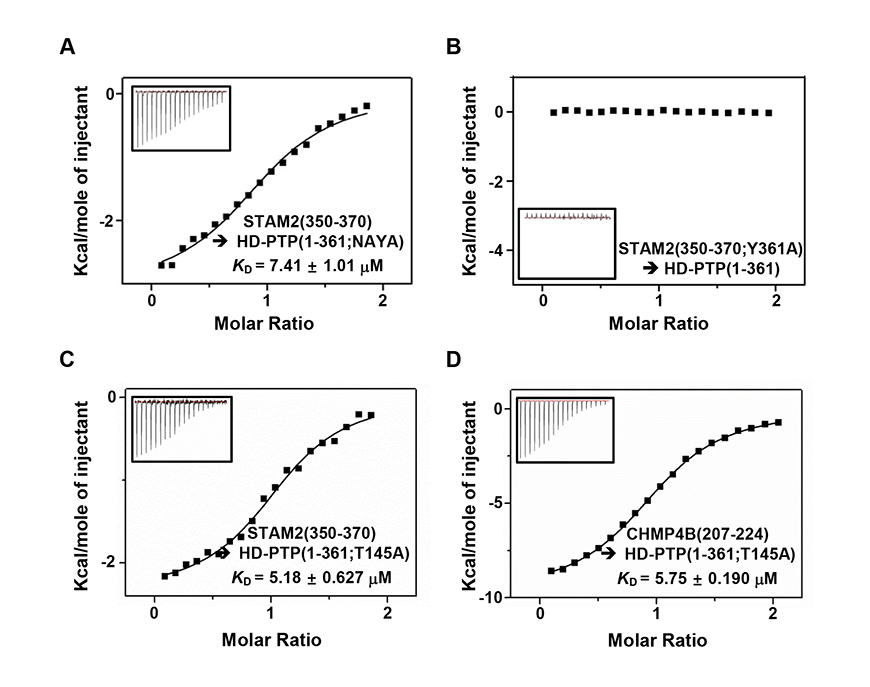

Supplement: S3 Fig — ITC measurements were carried out by titrating the 0.5 mM indicated peptide into the 50 μM wild type (B) or mutant (A, C-D) HD-PTP proteins. (TIF) [file pone.0149113.s003.tif]

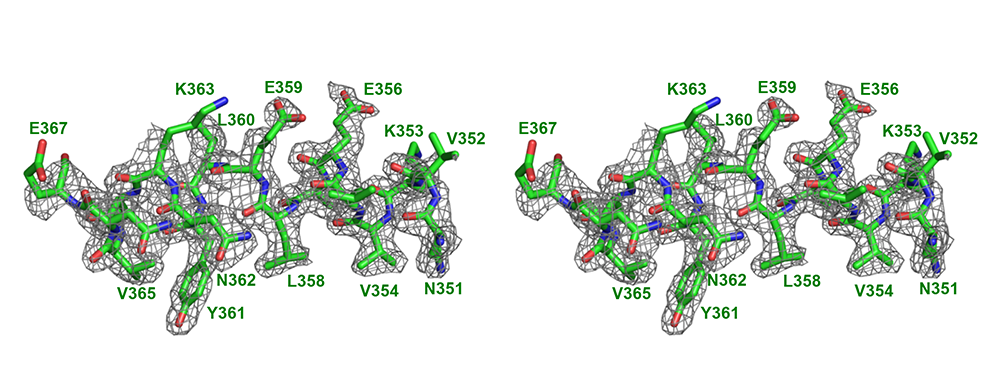

Supplement: S4 Fig — The STAM2(350–370) fragment in the Fig 2B is presented in sticks together with the 2Fo-Fc electron density omit map (grey mesh; contoured at 1.0 σ). (TIF) [file pone.0149113.s004.tif]

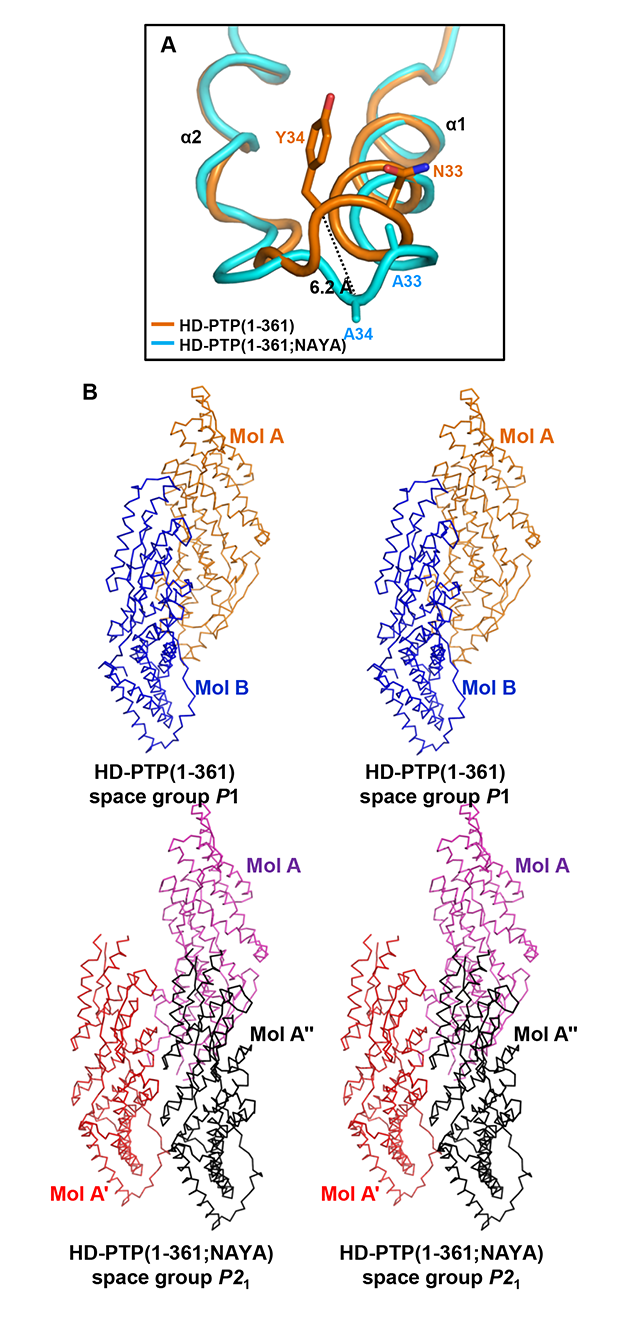

Supplement: S5 Fig — (A) Conformational change of HD-PTP induced by the introduction of two mutations. The two residues targeted for mutation are shown in sticks and are labeled. The dashed line indicates the movements of the Cα atoms of Tyr34 substituted to alanine. (B) Packing of HD-PTP molecules in crystals. HD-PTP(1–361) and HD-PTP(1–361;NAYA) are shown in Cα traces. For clarity, STAM2(350–370) is omitted. Mol A and B of HD-PTP(1–361) are in the same asymmetric unit; Mol A, A' and A'' of HD-PTP(1–361;NAYA) are not, but are symmetrically related. (TIF) [file pone.0149113.s005.tif]

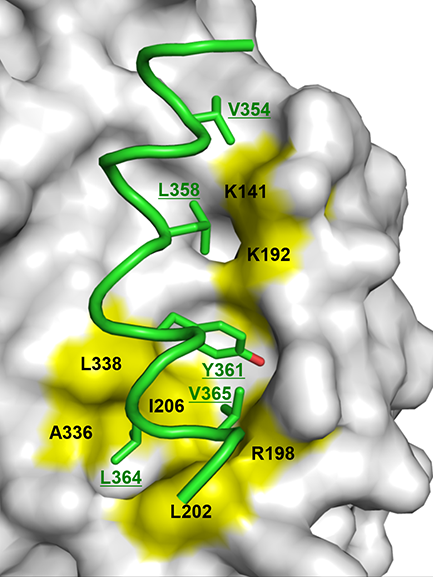

Supplement: S6 Fig — HD-PTP(1–361;NAYA) is represented in the surface model bound to the STAM2 fragment shown in green. Residues engaged in the intermolecular hydrophobic contacts (five from STAM2 and seven from HD-PTP; see Fig 2B) are labeled. Shown in stick representation are the labeled STAM2 residues; colored in yellow are the hydrocarbon portions of the side chain of the labeled HD-PTP residues. (TIF) [file pone.0149113.s006.tif]

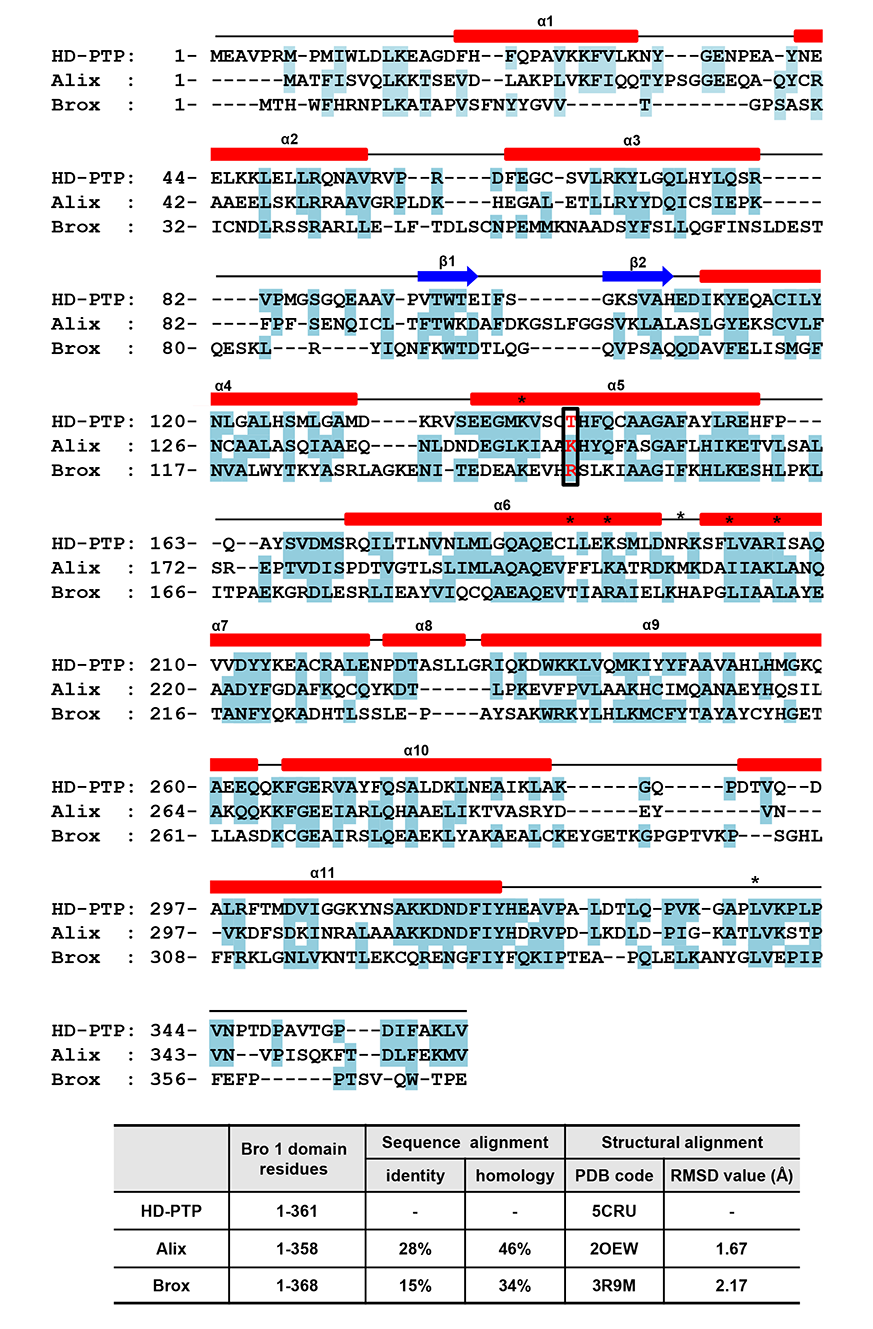

Supplement: S7 Fig — The sequences of the Bro1 domains of HD-PTP, Alix, and Brox are aligned based on a structural comparison. The secondary structures of HD-PTP are shown together. Conserved residues are shaded in cyan. The key residues adjusting STAM2 binding are shown in red and are highlighted by a black rectangle. Asterisks denote the HD-PTP residues involved in STAM2 binding. The sequence and structural alignment statistics are listed below. (TIF) [file pone.0149113.s007.tif]
